# Temporomandibular joint disorder from skull-base osteomyelitis: a case report

**DOI:** 10.1186/s40902-015-0041-1

**Published:** 2015-10-31

**Authors:** Suck-Chul Lee, Jae-Hyung Kim, Chul-Hoon Kim, Bok-Joo Kim

**Affiliations:** Department of Oral and Maxillofacial surgery, Dong-a University Hospital, Daesingongwon-ro 26, Seo-gu, Busan, 602-715 Korea

**Keywords:** Malignant external otitis, Skull-base osteomyelitis, Arthrocentesis with lavage

## Abstract

Skull-base osteomyelitis is a rare disease affecting the medulla of the temporal, sphenoid, and occipital bones. In general, it occurs due to external ear canal infections caused by malignant external otitis. Skull-base osteomyelitis usually affects elderly diabetic patients.

The patient, a 58-year-old man, was referred for evaluation and management of the left jaw. Clinical examination of the patient revealed pain in the left jaw and mouth-opening deflection to the left. The maximum active mouth opening was measured to about 27 mm. Panoramic, CT, and CBCT revealed bone resorption patterns in the left condyle. Through control of diabetes, continued pharmacological treatment, arthrocentesis, and occlusal stabilization appliance therapy were carried out. The extent of active mouth opening was increased to 45 mm, and pain in the left jaw joint was alleviated.

This was a case wherein complications caused by failure to control diabetes induced skull-base osteomyelitis. There is a need for continued discussion about the advantages and disadvantages of arthrocentesis with lavage for patients with skull-base osteomyelitis and other treatment options.

## Background

Malignant external otitis, though not a malignant tumor, is very invasive and difficult to resolve. Prior to the introduction of antibiotics, its mortality rate was 50 % [1, 2]. Malignant external otitis begins as external, necrotizing soft-tissue infection that can gradually spread, in severe cases, to cartilage and the base of the skull, the facial nerve, the trigeminal nerve, the optic nerve, the paranasal sinuses, and the temporomandibular joint. As clinical symptoms, earache, otorrhea, headache, and temporomandibular pain can be present; in severe cases, even facial nerve paralysis can occur. Malignant external otitis always remains localized in soft tissue; in cases where it spreads to the temporal bone or basal ganglia, it is known as skull-base osteomyelitis [3].

Skull-base osteomyelitis is a rare disease affecting the medulla of the temporal, sphenoid, and occipital bones. In general, it occurs due to external ear canal infections caused by malignant external otitis that proceeds to skull base and becomes chronic; however, atypical skull-base osteomyelitis that arises independently of any history of external otitis has been reported as well [3].

Skull-base osteomyelitis usually affects elderly diabetic patients. Diabetic patients have weak chemotaxis- or phagocytosis-functional cells such as polymorphonuclear leukocytes, monocytes, and macrophagocytes, and so are vulnerable to the bacteria causative of malignant external otitis [3]. Among such bacteria, pseudomonas aeruginosa is well known, though other strains also are detected; all, generally, are considered to be simple colony forming rather than pathogenic bacteria. Eumycetes, for example, sometimes infect patients with acquired immunodeficiency syndrome (AIDS) [4].

In the disease course, epidermis infection spreads to the dermis, causing an acute and chronic inflammatory reaction. Such infection spreads to the skull base, where it can be contracted to the infratemporal fossa, parotid gland, and cervical spine [5]. Generally this occurs unilaterally, but the spine can be infected by spreading of skull-base osteomyelitis to the opposite side. This is a serious disease with high mortality and multiple cranial paralysis rates [6].

With respect to pain and dysfunction, skull-base osteomyelitis manifests similarly to the initial symptoms of temporomandibular joint disorder. Thus, all too frequently, definitive diagnosis is delayed. We herein report, together with a review of the relevant literature, a clinical case of a patient who had been admitted to hospital for otorrhea, which was differentially diagnosed by skull-base osteomyelitis and improved by arthrocentesis.

## Case presentation

The patient, a 58-year-old man, was referred for evaluation and management of the left jaw joint during hospitalization in the Division of Otolaryngology due to an outbreak of malignant external otitis on November 6, 2012. He began treatment in otolaryngology mainly due to running ears. However, since his condition did not improve, incision and drainage of the left jaw joint were performed after hospitalization. Later, as the patient’s condition threatened to deteriorate after pharmacological treatment at another ENT hospital, he was sent to our tertiary care hospital for treatment. His medical history included diabetes that had resulted in diabetic neuropathy.

Clinical examination of the patient revealed pain in the left jaw and mouth-opening deflection to the left. The maximum active mouth opening was measured to about 27 mm, which limitation was due to continuous pain. Tender points in the left temporalis muscle and left jaw joint were noted.

**Fig. 1:**
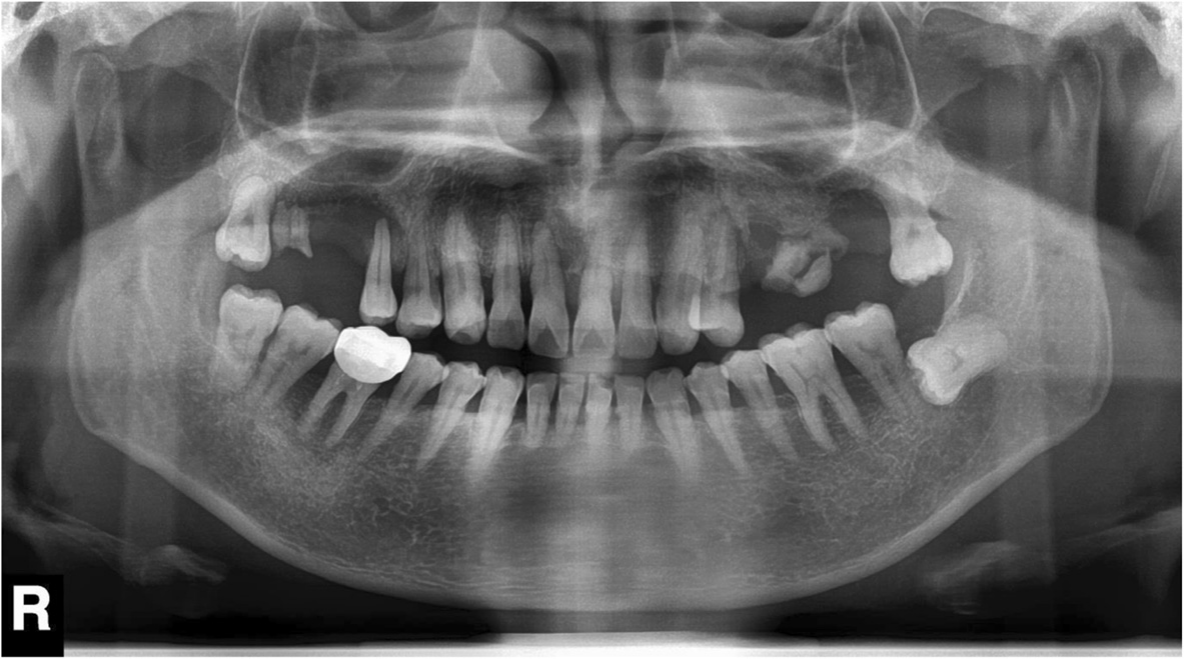
Panoramic radiographs of bone resorption patterns of left condyle

**Fig. 2:**
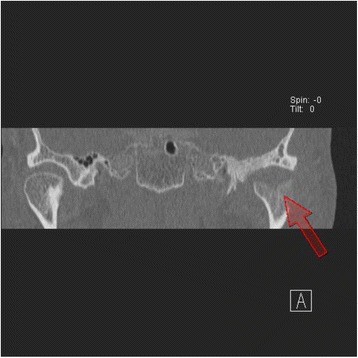
CT scans showing same bone resorption patterns of left condyle

**Fig. 3:**
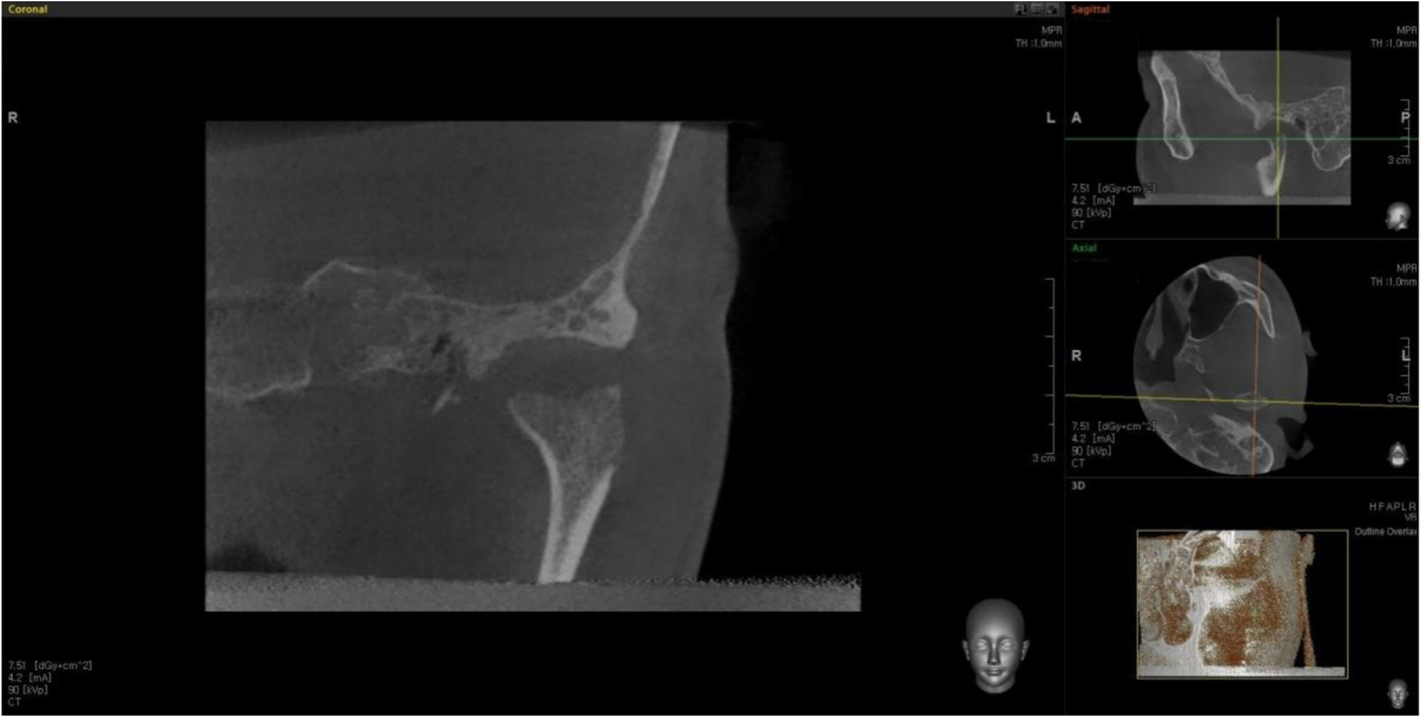
CBCT showing same bone resorption patterns of left condyle

**Fig. 4:**
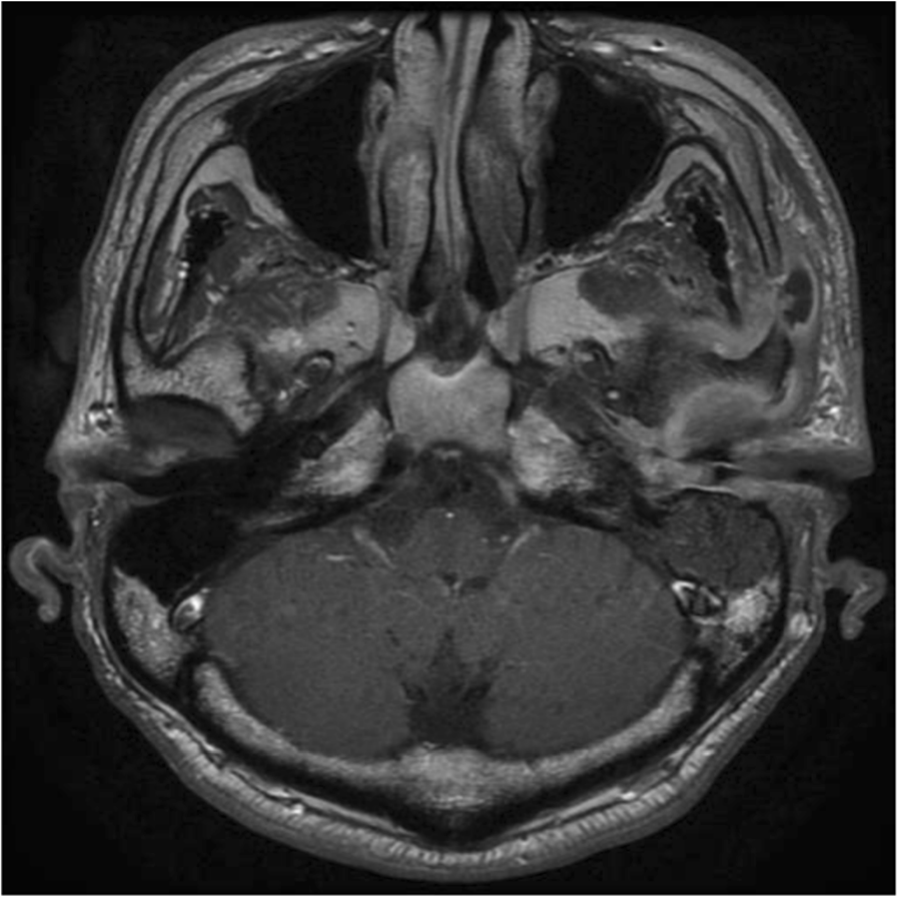
MRI reveals left temporomandibular joint surrounded by thickened soft tissue

**Fig. 5:**
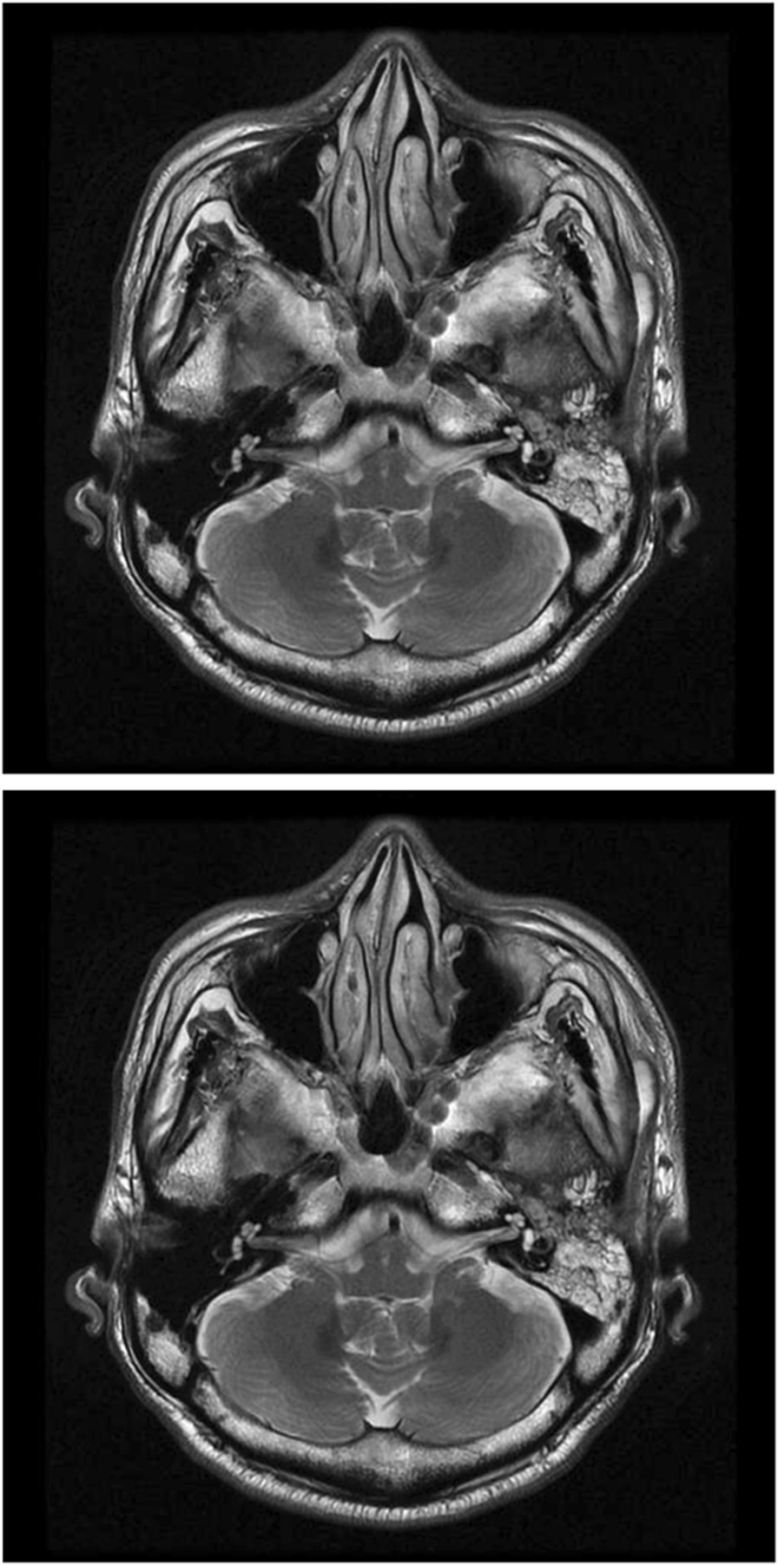
T2-weighted imaging showing effusion in left mastoid air cell

**Fig. 6:**
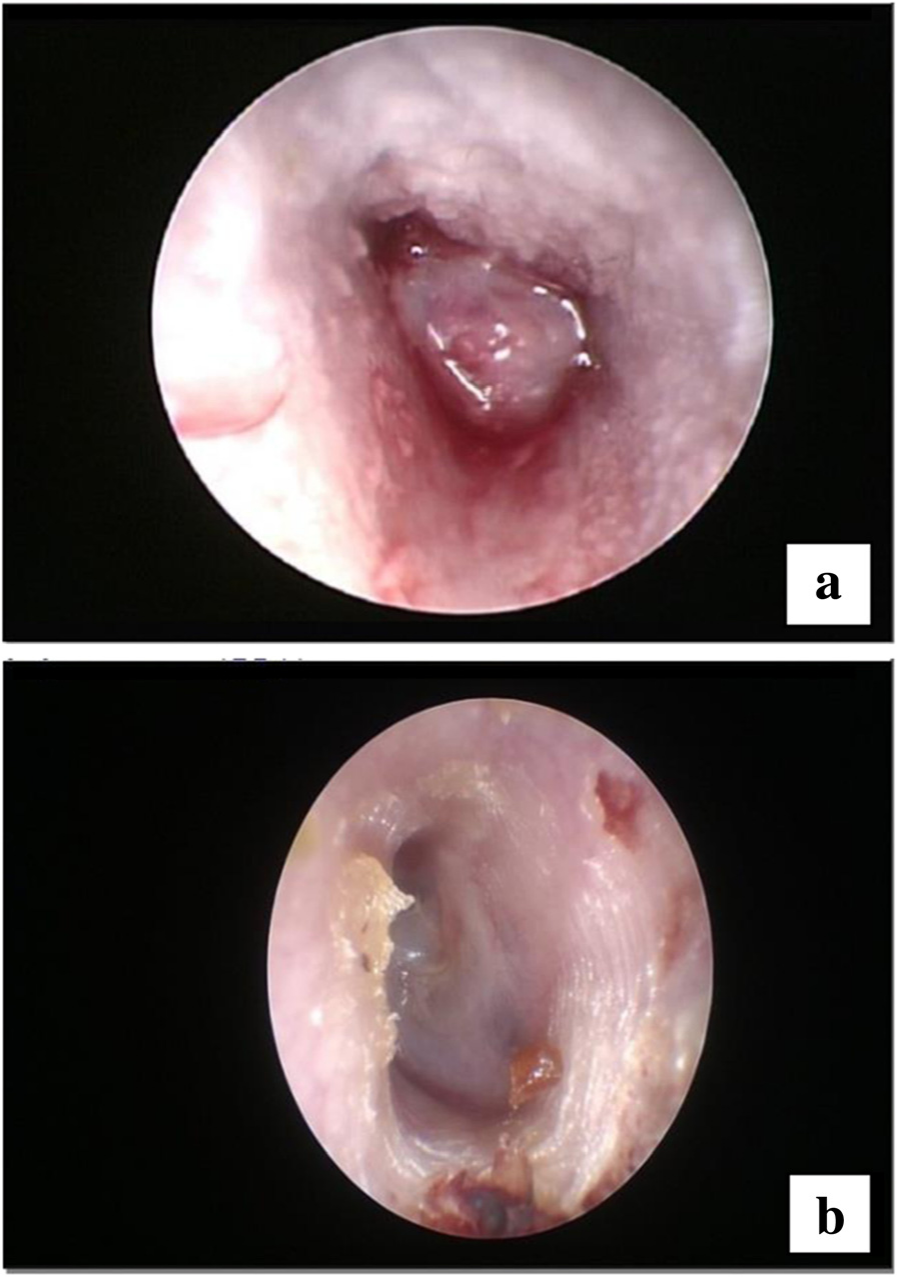
**a** Thickening of external auditory canal epithelium. **b** 7 months later

Panoramic radiographs revealed bone resorption patterns in the left condyle (Fig. 1). CT scans and cone beam computed tomography (CBCT) of the temporal bone showed the same patterns (Figs. 2 and 3). MRI findings revealed the left temporomandibular joint to be surrounded by thickened soft tissue (Fig. 4); T2-weighted imaging, meanwhile, showed effusion in the left mastoid air cell (Fig. 5). Based on the thickening of the external auditory canal epithelium (Fig. 6a, b), contrast-media imaging of diffusion with increased patterns around the thickening region, as well as discharge in the mastoid process, the patient was provisionally diagnosed with malignant external otitis, mastoiditis, and arthritis of the left temporomandibular joint. After treatment of the lesion by incision and drainage of the left jaw joint in the Department of Otorhinolaryngology, arthrocentesis and occlusal stabilization appliance therapy were carried out. Due to severe inflammation in the joint space, the arthrocentesis with lavage was performed twice. According to the results of a culture of the bacteria in the external auditory canal, carried out prior to incision and drainage, pseudomonas aeruginosa was detected; but according to the results of a bacterial culture implemented later, no bacteria were detected. After the arthrocentesis with lavage, the extent of active mouth opening was increased to 40 mm, and pain in the left jaw joint was alleviated.

**Fig. 7:**
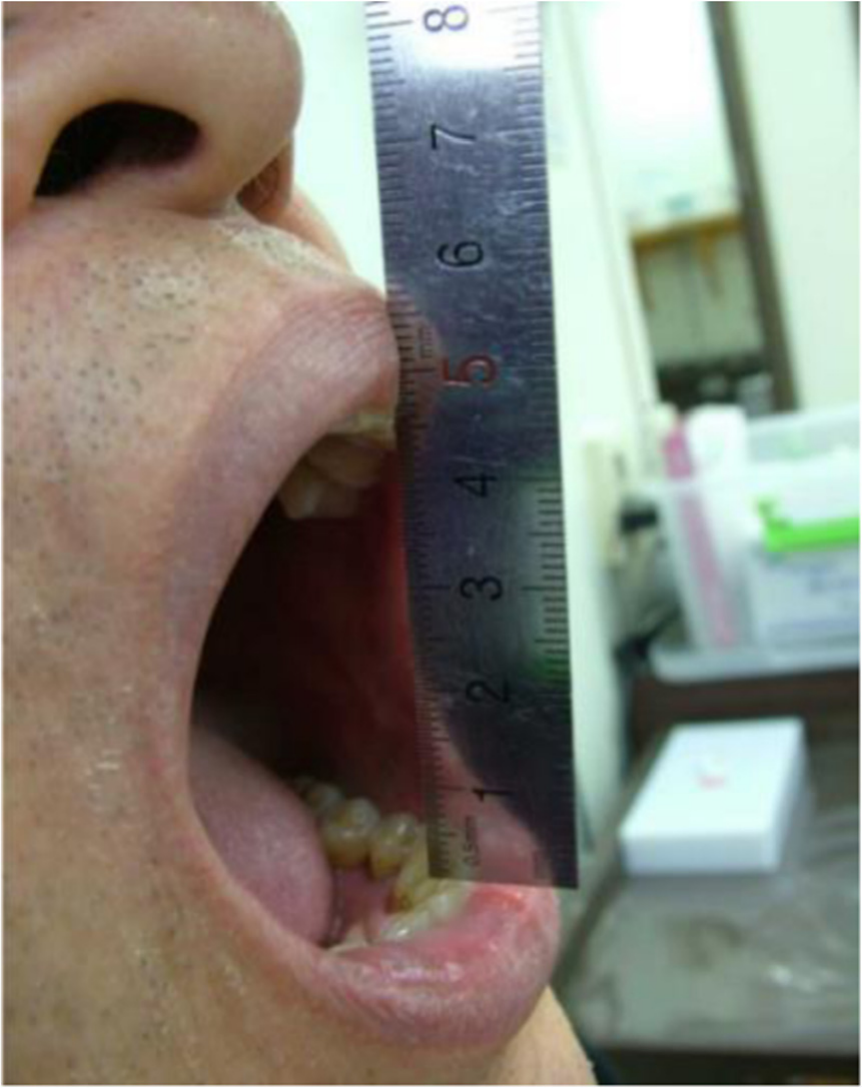
Extent of active mouth opening as increased to 45 mm

**Fig. 8:**
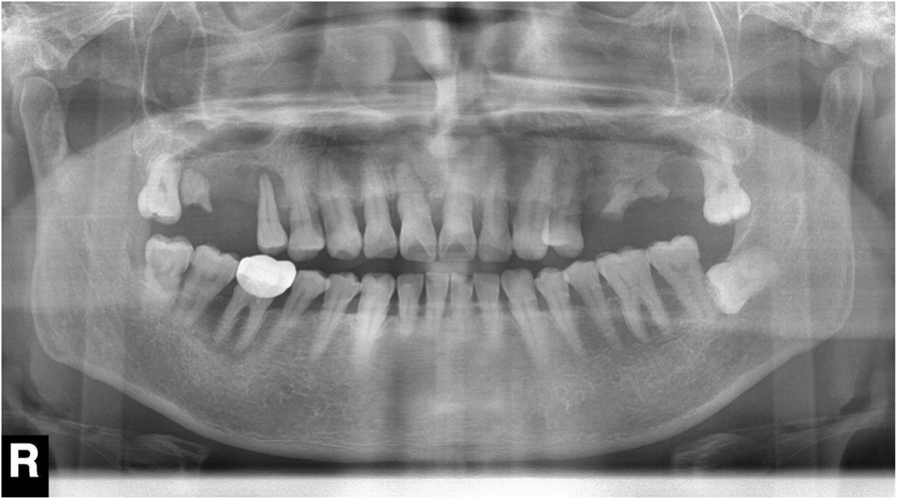
Panoramic images taken 1 year and 5 months after surgery, confirming significant reduction of bone resorption patterns in left condyle

**Fig. 9:**
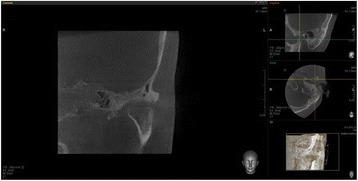
CBCT showing significant reduction of bone resorption pattern in left condyle

**Fig. 10:**
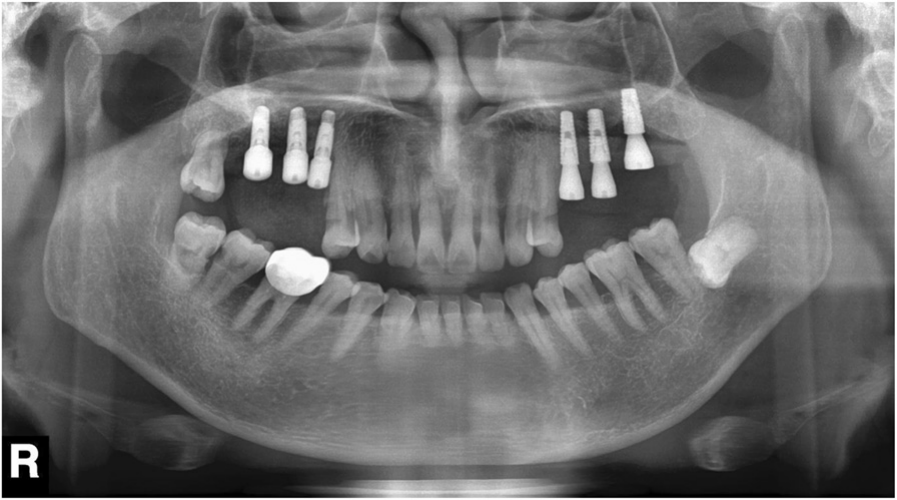
Panoramic images taken 2 years after surgery, confirming significant reduction of bone resorption patterns in left condyle

Approximately 1 month after the first arthrocentesis with lavage was performed, an occlusal stabilization appliance was employed, which effectively eliminated the pain in the left temporomandibular joint. The extent of active mouth opening was further increased, from 40 to 45 mm (Fig. 7). Then, close observation with regular examination and pharmacological treatment with quinolones such as ciprofloxacin were performed in our otorhinolaryngology clinic. Panoramic and CBCT images taken 1 year and 5 months after surgery confirmed that the bone resorption patterns in the left condyle had been significantly reduced (Figs. 8 and 9). Now, the patient, still pain-free in the left temporomandibular joint, currently is undergoing implantation treatment (Fig. 10).

### Discussion

Skull-base osteomyelitis arises mainly through secondary infection of malignant external otitis. Although skull-base osteomyelitis and malignant external otitis are considered to be identical concepts in many studies, it is more appropriate to conceive of and think about skull-base osteomyelitis with basal bone involvement in pathophysiological terms [3].

Important skull-base osteomyelitis risk factors include immunosuppression, diabetes, chronic mastoiditis, sinusitis, malignant external otitis, infectious disease treated poorly with antibiotics, and decreased immunity due to the use of steroids or infection with human immunodeficiency virus [7]. The most common disease associated with patients hospitalized due to skull-base osteomyelitis is diabetes [8]. Skull-base osteomyelitis most typically infects the diabetic elderly; accordingly, infection caused by chronic external otitis occurs secondarily in connection with temporal bone [9]. However, atypical skull-base osteomyelitis, which arises in the absence of any history of external otitis, has only rarely been reported [10]. Skull-base osteomyelitis is an often fatal disease, with mortality rates ranging from 28 to 60 % along with cranial nerve palsy in cases of delayed diagnosis and treatment [11]. Pseudomonas aeruginosa is the most common (99.2 %) cause of skull-base osteomyelitis, whereas fungal skull-base osteomyelitis, caused by aspergilus and candida, is very rarely observed [12].

The most important consideration in the treatment of malignant external otitis is the control of diabetes and its complications. The excision of lesions is not generally recommended, though there is a need to remove locally inflamed tissues and bacteria. Generally, treatment is performed with antibiotic-mediated therapy using penicillin, ciprofloxacin and third-generation cephalosporin, which approach helps to reduce the mortality rate by 0–15 % [13]. In cases where the middle cranial fossa and foramen magnum are contracted, aspergilus infection occurs, from which poor-prognosis facial paralysis arises [14, 15].

According to the literature, surgical removal of the temporomandibular joint remains controversial. Long-term antibiotic therapy might affect the same results, even without other surgical procedures, and non-invasive treatment such as arthrocentesis with lavage also is recommended [16]. However, surgical treatment is recommended in cases where an abscess has formed in the joint space, or where extensive bone destruction of the glenoid and condyle is observed [17].

As for typical cases, conservative treatment primarily is performed when malignant external otitis affects the temporomandibular joint. However, in the present investigation, panoramic images showed cortical erosion of the upper mandibular condyle, and an MRI revealed condylar locking, mouth-opening limitation, and inflammation within the joint space. For this patient, arthrocentesis with lavage was performed.

## Conclusions

This was a case wherein complications caused by failure to control diabetes induced skull-base osteomyelitis, which was accompanied by an infected jaw joint, ear pain, otorrhea resulting from external ear canal pathology on the affected side, and mouth-opening limitations. Through control of diabetes, continued pharmacological treatment and arthrocentesis with lavage, we could achieve a rapid increase in the maximum mouth opening. For future work, there is a need for continued discussion about the advantages and disadvantages of arthrocentesis with lavage for patients with skull-base osteomyelitis and other treatment options.

## Consent

Written informed consent was obtained from the patient for the publication of this report and any accompanying images.

## Abbreviations

CBCT: cone beam computed tomography
CT: computed tomography
MRI: magnetic resonance imaging
